# Contents of lobetyolin, syringin, and atractylolide III in *Codonopsis pilosula* are related to dynamic changes of endophytes under drought stress

**DOI:** 10.1186/s13020-021-00533-z

**Published:** 2021-11-22

**Authors:** Yichuan Liang, Guangfei Wei, Kang Ning, Guozhuang Zhang, Youping Liu, Linlin Dong, Shilin Chen

**Affiliations:** 1 School of Pharmacy, Chengdu University of Traditional Chinese Medicine, Chengdu, 611137 China; 2 Key Laboratory of Beijing for Identification and Safety Evaluation of Chinese Medicine, Institute of Chinese Materia Medica, China Academy of Chinese Medical Sciences, No. 16 Nanxiaojie, Dongzhimennei Ave., Beijing, 100700 China

**Keywords:** *Codonopsis pilosula*, Endophytes, Pharmacodynamic compounds, Drought stress

## Abstract

**Background:**

*Codonopsis pilosula*, an important medicinal plant, can accumulate certain metabolites under moderate drought stress. Endophytes are involved in the metabolite accumulations within medicinal plants. It is still unknown that the endophytes of *C. pilosula* are associated with the accumulations of metabolites. This study aims to investigate the promoting effect of endophytes on the accumulations of active substances in *C. pilosula* under drought stress.

**Methods:**

High–performance liquid chromatography and high–throughput sequencing technology were performed to investigate changes in the contents of secondary metabolite and endophyte abundances of *C. pilosula* under drought stress, respectively. Spearman’s correlation analysis was further conducted to identify the endophytic biomarkers related to accumulations of pharmacodynamic compounds. Culture-dependent experiments were performed to confirm the functions of endophytes in metabolite accumulations.

**Results:**

The distribution of pharmacological components and diversity and composition of endophytes showed tissue specificity within *C. pilosula*. The contents of lobetyolin, syringin, and atractylolide III in *C. pilosula* under drought stress were increased by 8.47%‒86.47%, 28.78%‒230.98%, and 32.17%‒177.86%, respectively, in comparison with those in untreated groups. The Chao 1 and Shannon indices in different parts of drought–stressed *C. pilosula* increased compared with those in untreated parts. The composition of endophytic communities in drought treatment parts of *C. pilosula* was different from that in control parts. A total of 226 microbial taxa were identified as potential biomarkers, of which the abundances of 42 taxa were significantly and positively correlated to the pharmacodynamic contents. Culture-dependent experiments confirmed that the contents of lobetyolin and atractylolide III were increased by the application of *Epicoccum thailandicum*, *Filobasidium magnum*, and *Paraphoma rhaphiolepidis* at the rates of 11.12%‒46.02%, and that the content of syringin was increased by *Pseudomonas nitroreducens* at the rates of 118.61%‒119.36%.

**Conclusions:**

Certain endophytes participated in the accumulations of bioactive metabolites, which provided a scientific evidence for the development and application of microorganisms to improve the quality of traditional Chinese medicine.

**Supplementary Information:**

The online version contains supplementary material available at 10.1186/s13020-021-00533-z.

## Introduction

Medicinal plants are indispensable to improve human health, more than 70% of the population in developing countries relies on herbal medicine to prevent and treat diseases [1]. Codonopsis Radix, the dried root of *Codonopsis pilosula*, is a common traditional Chinese medicine, and renowned for remarkable effect on improving gastrointestinal function, nourishing the spleen and lung, enhancing immunity, and delaying senescence [2]. This medicine is a widely used well–*qi*–tonifying medicine of homology of medicine and food, and its comprehensive output value is more than $1.5 billion annually [3, 4] Importantly, the pharmacological actions of Codonopsis Radix are attributed to its bioactive metabolites, such as lobetyolin, syringin, angelicin, and atractylolide III [5]. These active compounds could regulate gastrointestinal motility, protect gastric mucosa, lower blood glucose level, and improve Alzheimer’s disease [6–8]. Due to the specific ecological requirements, *C. pilosula* is mainly cultivated in Gansu Province, China, where the climatic condition is an arid and semiarid temperate continental climate [4]. Environmental stress could stimulate medicinal plants to synthesize more secondary metabolites to adapt to adversity, thus, drought is conducive to the accumulations of secondary metabolites in *C. pilosula* [4]. For example, compared with the untreated root part, the roots of *C. pilosula* under drought treatment contained remarkably increased syringin [9]. Research on the accumulations of secondary metabolites of *C. pilosula* in response to drought stress and elucidating the underlying mechanism are important for quality improvements and good agriculture managements.

Endophytes play an important role in the accumulations of secondary metabolites within medicinal plants [10]. Moderate water deficit could lead to changes in diversity, composition and function of endophytes, and intensify the plant-endophyte interactions which might increase the accumulations of secondary metabolites in medicinal plants [10]. During the process of environment stress, endophytes are stimulated to produce biologically active compounds, including terpenoids, alkaloids, flavonoids, and phenylpropanoids [11]. The endophytic *Paenibacillus polymyxa*, isolated from *Panax ginseng* leaf, could synthesize ginsenosides under drought stress [12, 13]. Endophytes could also participate in the transformations of active secondary metabolites in medicinal plants [14]. *Aspergillus fumigatus* purified from *Artemisia annua* L*.* converted arteannuic acid into artemisinin with peroxide bridge structure for anti–malarial effects [14]. Besides, endophytes could stimulate medicinal plants to produce secondary metabolites through endophyte–plant interactions under environmental stress [10]. For instance, the endophytic *Pseudomonas fluorescens* ALEB7B could stimulate *Atractylodes lancea* to generate reactive oxygen species (ROS) to increase sesquiterpenoid content; *Gilmaniella* sp. AL12, an endophytic fungus, could induce *A. lancea* to produce volatile oils, such as β–caryophyllene, zingiberene, caryophyllene, hinesol, β–eudesmol, and atractylone [15, 16]. These researches indicated that endophytes participated in the accumulations of secondary metabolites in medicinal plants. Endophytes become an important source of active substance to meet the increasing demands of clinical medicines [10]. However, comprehensive reports on the correlation between endophyte changes and accumulations of secondary metabolites under drought stress in *C. pilosula* are few. Thus, identification of strains involving in the accumulations of secondary metabolites contributed to investigate the interactions and associations between endophytes and secondary metabolism.

Here, the increase in the contents of lobetyolin, syringin, and atractylolide III under drought treatment was hypothesized to be related to endophyte changes in *C. pilosula*. Dynamic changes in the endophytes and pharmacodynamic compounds in different parts of *C. pilosula* under drought treatment were investigated through high–throughput sequencing and high–performance liquid chromatography (HPLC), respectively. The correlations between endophyte changes and accumulations of secondary metabolites were analyzed to confirm the potential biomarkers related to the target compounds*.* And, culture-dependent experiments were conducted to verify the functions of identified endophytes in metabolite accumulations.

## Materials and methods

### Experimental design and sample collection

Pot experiments with different moisture contents in soil were conducted under greenhouse conditions on a 16 h/8 h light/dark cycle at 22 °C ± 2 °C [17]. In brief, 1–year–old *C. pilosula* seedlings were collected from Weiyuan County, Gansu Province in China, and transplanted into pots (height: 20 cm; diameter: 20 cm). Each pot contained 10 kg of sandy loam from the local farmland. The moisture content (18%–20%) of the sandy loam measured by oven–drying method was used as untreated groups (CK) [18]; 10%–12% moisture content in sandy loam was used as the drought treatment groups (DT) [19]. After *C. pilosula* seedlings were transplanted, the moisture content in the sandy loam was maintained at the control level (18%–20%) for 4 weeks. During the experimental stage, the moisture content of sandy loam was measured by weighing method [20]. There were three biological repetitions, and each repetition contained three pots. The pots were periodically moved to minimize the effects of environmental heterogeneity [20].

Plant samples from DT and CK groups were collected at the first, third, and eighth days of drought treatment. The samples were washed with distilled water and separated into three different parts (leaf, stem, and root). The plant parts were successively immersed in 75% ethanol (v/v) for 30 s and 0.1% mercuric chloride for 5 min, and then rinsed six times with sterile water for surface sterilization [21]. To verify if the plant samples were surface sterilized, 250 µL of the last wash water was coated on nutrient agar (NA) medium (composed of tryptone 10.0 g, beef extract 3.0 g, sodium chloride 5.0 g, agar 10.0 g and pH 7.0–7.3 in 1 L water) and potato dextrose agar (PDA) medium (composed of potato 220 g, glucose 15 g, and agar 10 g in 1 L water), and cultured at 37 °C and 25 °C for 8 days, respectively [22]. The total samples were frozen immediately in liquid nitrogen and stored at − 80 °C for chemical and molecular analyses [22, 23].

### HPLC–UV analysis

Lobetyolin, syringin, and atractylolide III standards (purity ≥ 98.0%; batch numbers: CHB180224, CHB180530, and E1708006, respectively) were purchased from Chengdu Croma Biotechnology Co., Ltd (Chengdu, China). Approximately 0.1 g of powder each part was extracted with 1.5 mL of methanol under ultrasound for 40 min. After centrifugation at 4000×*g* for 10 min, the supernatant was filtered by 0.45 μm microporous membrane [9]. All samples were analyzed by an Agilent HPLC–UV 1260 series system (Agilent, USA) equipped with a quaternary pump, automatic sampler, column compartment, and variable wavelength detector. The chromatogram column was a C_18_ column (4.6 mm × 250 mm, 5 µm; Eclipse XDB; Agilent, USA), the flow rate was 1.0 ml min^−1^, the column temperature was 25 °C, and the injection volume was 20 μL. Detection wavelengths were 267 nm (lobetyolin and syringin) and 220 nm (atractylolide III), and the mobile phase was composed of acetonitrile (A) and water containing 0.1% phosphoric acid (B). The gradient elution conditions were set as follows: 0–10 min, 5%–15% A; 10–20 min, 15% A; 20–35 min, 15%–45% A; 35–40 min, 45%–85% A; 40–45 min, 85%–5% A; and 45–50 min, 5% A [9]. The validation results indicated that the reference HPLC–UV method had good peak separation, accurate quantification, and high durability (Additional file 1).

### High–throughput sequencing and bioinformatics analysis of microbiome

Total DNA was extracted from the three different parts by using the FastDNA SPIN Kit (MP Biomedicals, Santa Ana, CA, USA). The V4-V5 region of bacterial 16S rRNA gene was amplified using primers 515F (5′–GTGCCAGCMGCCGCGG–3′) and 907R (5′–CCGTCAATTCMTTTRAGTTT–3′) [24]. The internal transcribed spacer (ITS) region of fungi was amplified with primers ITS1–F (5′–CTTGGTCATTTAGAGGAAGTAA–3′) and ITS2–R (5′–GCTGCGTTCTTCATCGATGC–3′) [25]. Amplification, purification, and generation of sequencing library were performed as previous reports [26]. The library was subjected to paired–end sequencing on an Illumina MiSeq PE300 platform (Majorbio Company, Shanghai, China). All raw sequences were uploaded to the National Center for Biotechnology Information (NCBI; accession numbers: leaf, PRJNA721564; stem, PRJNA721933; root, PRJNA663111). Low–quality reads (below the quality score of 20) were deleted [27]. Next, paired–end reads with at least 30 bp overlap were merged by FLASH (v2.4.0) software to obtain sequences; then, the sequences were assigned to different samples on the basis of unique barcode [28]. Usearch (v10) was performed to remove chimeras and cluster operational taxonomic units (OTUs) with 97% similarity cutoff. α–diversity indices, including Chao 1 and Shannon (*H'*) indices, were calculated using QIIME (v1.9.0) [24]. Principal coordinate analysis (PCoA) based on weighted UniFrac distance was performed to determine the significant differences among different groups [29]. Linear discriminant analysis Effect size (LefSe, v1.9.0) was conducted to characterize the features that differentiated the microbial communities [30]. Then, Spearman’s correlation analysis was used to analyze the relationships between the contents of pharmacological components and the abundances of microbial biomarkers identified by LefSe [24].

### Analysis of pharmacodynamic substance accumulation

According to the results of endophytic biomarkers obviously correlated to the contents of pharmacodynamic compounds, we screened and obtained the strains from the microbiological culture collection library in our laboratory [31]. The raw sequences of identified strains were uploaded to NCBI (Accession number: PRJNA 667812). The strains from the following species, namely *Bacillus flexus*, *Pseudomonas nitroreducens*, *Pseudomonas entomophila*, *Paraphoma rhaphiolepidis*, *Plectosphaerella niemeijerarum*, *Alternaria alstroemeriae*, *Epicoccum thailandicum*, *Filobasidium magnum*, *Fusarium pseudoanthophilum*, and *Fusarium solani*, were used to confirm their functions in metabolite accumulations. Sterile *C. pilosula* powder (steam sterilization at 120 ℃ for 20 min) were used as carbon source to determine the influences of the strains on metabolite accumulations [32]. 4 mL of sterile liquid medium (NA medium without agar for endophytic bacteria and PDA medium without agar for endophytic fungi) with 0.1 g of sterile *C. pilosula* powder was used for the untreated groups. For the treatment groups, 2 mL of endophyte solution, 2 mL of sterile liquid medium, and 0.1 g of sterile *C. pilosula* powder were used. In addition, 2 mL of sterile liquid medium and 2 mL of endophyte solution (endophyte + medium) were utilized for the groups used to verify whether the microorganism itself could produce pharmacodynamic substances. After 9 days of culture (170 rpm min^−1^, 37 °C for bacteria and 28 °C for fungi), the culture solutions were added with 500 µL n–butanol solution to stop the reaction and concentrated in vacuum until dryness; then, extraction and quantitative analysis of lobetyolin, syringin, and atractylolide III in residues were carried out as previously described [9]. The increase rates of the pharmacodynamic substances were calculated as follows: increase rate of pharmacodynamic substance = (the concentration of pharmacodynamic substance in treatment group−the concentration of pharmacodynamic substance in untreated group)/(the concentration of pharmacodynamic substance in untreated group) × 100% [32].

## Results

### The effects of drought stress on the productions of lobetyolin, syringin, and atractylolide III in different parts of *C. pilosula*

**Fig. 1:**
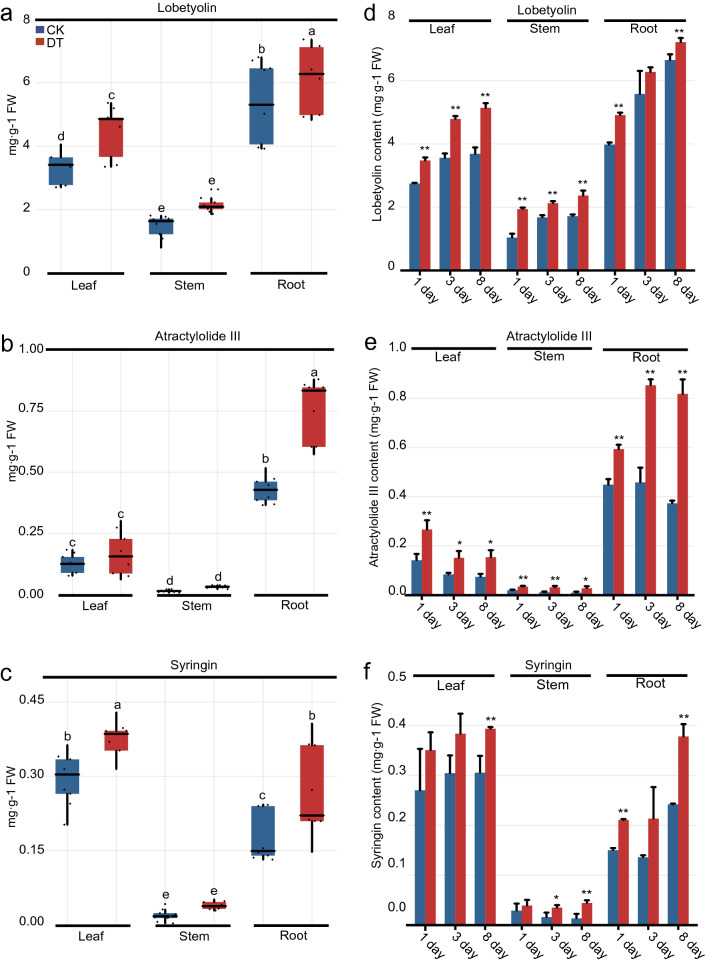
Effects of drought stress on the productions of lobetyolin, syringin, and atractylolide III in different parts of *C. pilosula.* **a**–**c** Contents of lobetyolin, atractylolide III and syringin in different parts of *C. pilosula*, respectively. Different letters indicate significant difference by Duncan’s multiple range test (*P* < 0.05). **d–f** Lobetyolin, atractylolide III and syringin contents in *C. pilosula* under drought stress, respectively. *CK* untreated group, *DT* drought treatment group, *FW* fresh weight. Columns represent the means of three biological repeats ± standard deviation (SD). Asterisks denote significant difference based on Student’s t-test (**P < 0.01; *P < 0.05)

The contents of lobetyolin, syringin, and atractylolide III in different parts of *C. pilosula* were detected through HPLC–UV analysis. According to Duncan’s multiple range test, the average contents of lobetyolin and atractylolide III were significantly high in the root part at 1–8 days (5.77 and 0.59 mg·g^−1^, respectively), in comparison with those in the leaf (3.90 and 0.15 mg·g^−1^, respectively) and stem parts (1.81 and 0.02 mg g^−1^, respectively; Fig. 1a, b). Besides, the average contents of lobetyolin, syringin, and atractylolide III were significantly lower in the stem part at 1–8 days (1.81, 0.03, and 0.02 mg·g^−1^, respectively) than that of the leaf (3.90, 0.34, and 0.15 mg·g^−1^, respectively) and root parts (5.77, 0.22, and 0.59 mg·g^−1^, respectively; Fig. 1a–c). Based on Student’s t–test, the lobetyolin contents in root (except for the third day), stem, and leaf parts of *C. pilosula* subjected to drought treatment for 1, 3, and 8 days markedly increased by 8.47%–23.40%, 26.67%–86.47%, and 26.41%–39.42%, respectively, in comparison with the untreated parts (Fig. 1d). Meanwhile, the atractylolide III content in the root, stem, and leaf parts under drought treatment for 1–8 days was remarkably increased by 32.17%–119.17%, 74.43%–177.86%, and 80.51%–107.50%, respectively, in comparison with those in control parts (Fig. 1e). In addition, the data indicated that the syringin content in the root, stem, and leaf parts after 8 days of drought treatment was observably increased by 56.30%–58.31%, 229.61%–230.98%, and 28.78%–31.31%, respectively, compared with that of the untreatment (Fig. 1f). Collectively, these results indicated that the accumulations of lobetyolin, syringin, and atractylolide III showed tissue specificity in *C. pilosula*, and their contents were increased in drought–stressed tissues.

### The effects of drought stress on the alpha diversity of endophytes in different parts of *C. pilosula*

**Fig. 2:**
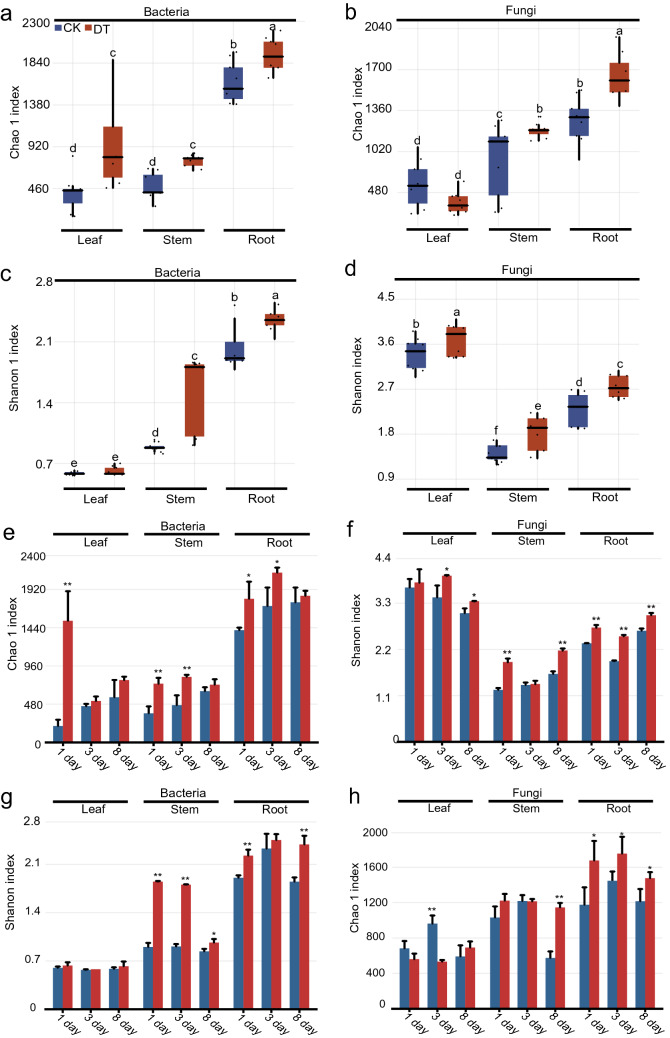
Effects of drought stress on the Chao 1 and Shannon indices of endophytic communities in different parts of *C. pilosula.* **a**–**d** Chao 1 (**a** and **b**) and Shannon (**c** and **d**) indices of endophytic communities in different parts of *C. pilosula*. Different letters indicate significant difference by Duncan’s multiple range test (*P* < 0.05). **e**–**h** Chao 1 (**e** and **h**) and Shannon (**f** and **g**) indices of endophytic communities in *C. pilosula* under drought stress. CK, untreated group; DT, drought treatment group. Columns represent the means of three biological repeats ± SD. Asterisks indicate significant difference based on Student’s t–test (**P < 0.01; *P < 0.05)

A total of 1,057,465, 1,140,973, and 1,121,510 16S rRNA reads, as well as 1,082,687, 1,131,502, and 1,078,567 ITS reads were obtained from the leaf, stem, and root tissues, respectively (Additional file 2: Table S1–S3). The mean sequencing depth per sample was 62,820 for bacteria and 60,977 for fungi, respectively. Chao 1 index showed a higher diversity number of microbial species in the roots than that of leaves and stems (Duncan’s multiple range test, *P* < 0.05; Fig. 2a, b and Additional file 3). *H’* index revealed that the diversity of endophytic bacteria in the roots was the highest, followed by those in the stem and leaf parts, whereas the diversity of endophytic fungi was the highest in the leaf part, followed by the root and stem parts (Duncan’s multiple range test, *P* < 0.05; Fig. 2c, d and Additional file 3). The bacterial Chao 1 index was higher in the leaf, stem, and root parts under 1-day-drought treatment than in the control parts (Student’s t-test, *P* < 0.05; Fig. 2e and Additional file 3). The fungal *H'* index in the drought treatment groups remarkably increased after 8 days compared with that of the untreated groups (Student’s t-test, *P* < 0.05; Fig. 2f and Additional file 3). In addition, the Chao 1 and *H'* indices of other drought treatment parts in *C. pilosula* (except for the Chao 1 of fungi in leaf part) displayed an increasing trend, or were markedly increased in comparison with those in the untreated parts (Fig. 2e–h and Additional file 3). These results revealed that the alpha diversity of endophytes in *C. pilosula* was tissue-dependent, and that the drought treatment could increase the alpha diversity of endophytes.

### The effects of drought stress on the beta diversity of endophytes in different parts of *C. pilosula*

**Fig. 3:**
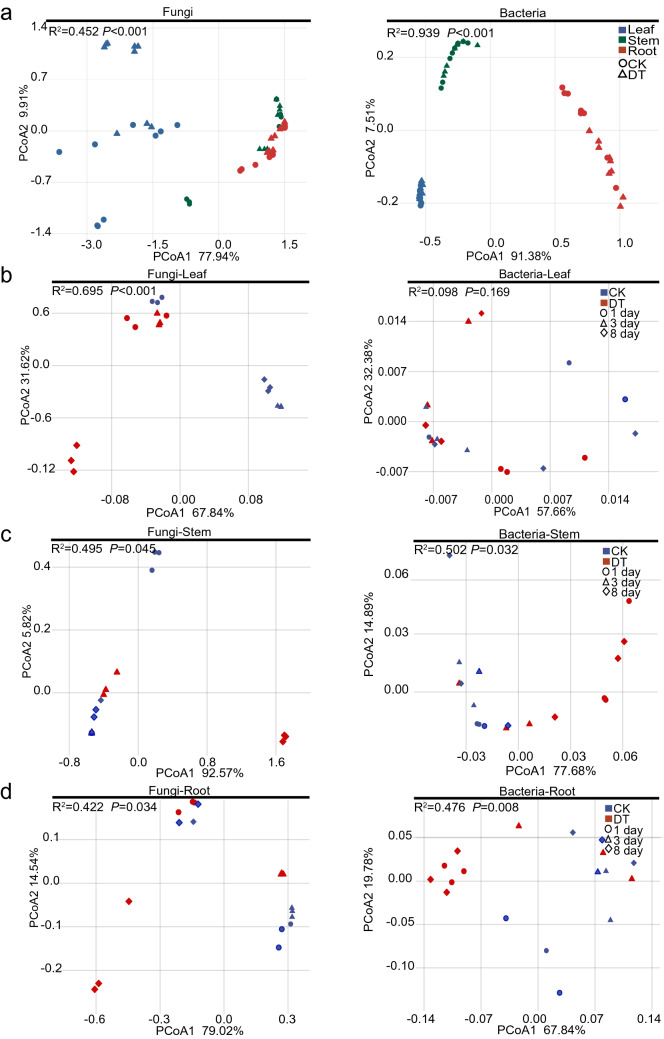
PCoA plot of endophytic communities in *C. pilosula.* **a** PCoA of endophytic communities in different parts of *C. pilosula*. **b**–**d** PCoA of endophytic communities in leaf, stem, and root parts between the untreated and drought treatment groups, respectively. *CK* untreated group, *DT* drought treatment group, *PCoA* principal coordinate analysis. PCoA based on the weighted UniFrac matrix

PCoA based on weighted UniFrac distances revealed that the microbial communities were remarkably different between different parts of *C. pilosula* (bacteria, R^2^ = 0.939, *P* < 0.001; fungi, R^2^ = 0.452, *P* < 0.001; Fig. 3a and Additional file 3). The PCoA results showed that the endophytic communities in *C. pilosula* parts subjected to drought treatment were significantly different from those of the control parts. The first and second principal component axes explained 67.84% and 31.62% of the total fungi changes in the leaf part (R^2^ = 0.695, *P* < 0.001; Fig. 3b and Additional file 3: Dataset S1). The bacterial communities in the leaf parts were clustered and negligible change (R^2^ = 0.098, *P* = 0.169; Fig. 3b and Additional file 3: Dataset S2). Moreover, the first (92.57% contribution) and second (5.82% contribution) principal component axes differentiated the fungal communities in drought treatment and untreated stem parts (R^2^ = 0.495, *P* = 0.045; Fig. 3c and Additional file 3: Dataset S3). The first principal component (77.68% contribution) distinguished the bacterial communities in the stem part treated by drought stress for 1 and 8 days from those in the untreated counterpart (R^2^ = 0.502, *P* = 0.032; Fig. 3c and Additional file 3: Dataset S4). Furthermore, the first principal component (79.02% contribution) differentiated the fungal communities in the root part under drought treatment for 1 and 8 days from those in the control treatment for 1 and 3 days; The second principal component (14.52%) highlighted the fungal communities in the root part subjected to drought treatment for 3 days from those in untreated groups (R^2^ = 0.422, *P* = 0.034; Fig. 3d and Additional file 3: Dataset S5). The first principal component axis (67.84% contributions) indicated that in the 1 and 8 days of experiment, the bacterial communities in the drought-treated roots were distinctly different from those of the control parts (R^2^ = 0.476, *P* = 0.008; Fig. 3d and Additional file 3: Dataset S6). These results demonstrated that the beta diversity of endophytic communities in *C. pilosula* displayed tissue specificity and remarkable differences between untreated (except for bacteria in the leaf compartment) and drought treatment tissues.

### The effects of drought stress on the compositions of endophytes in different parts of *C. pilosula*

**Fig. 4:**
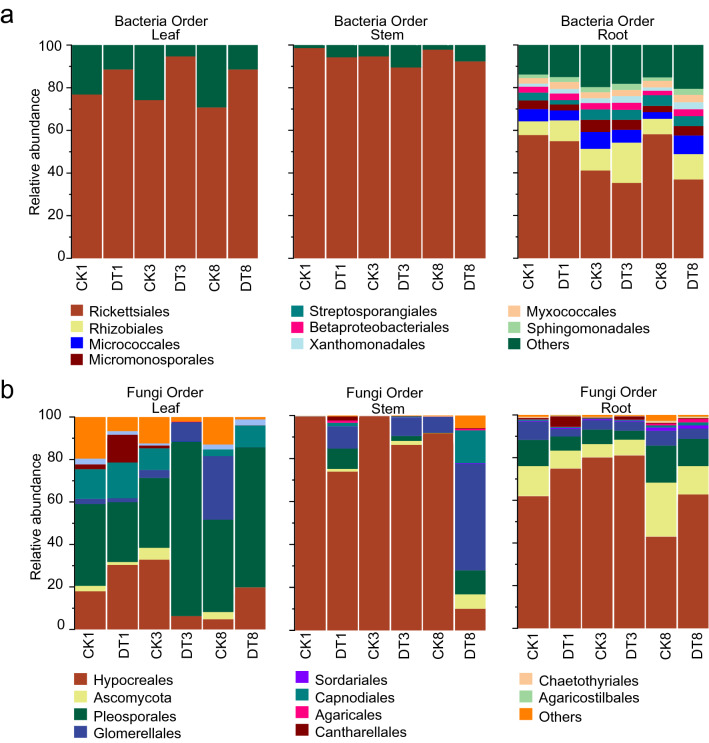
Effects of drought stress on the comositions of endophytes in different parts of *C. pilosula.* **a**, **b** Composition of bacterial and fungal microbiomes in different parts of *C. pilosula*, respectively. CK, untreated group; DT, drought treatment group. DT1, DT3, and DT8 represent DT on days 1, 3, and 8 of drought stress, respectively; CK1, CK3, and CK8 represent CK on days 1, 3, and 8 of drought stress, respectively

The composition of endophytic communities in different parts of *C. pilosula* showed remarkable discrepancies (Fig. 4 and Additional file 3). The bacterial communities in different parts of *C. pilosula* at the order level were dominated by the bacterial OTUs of Rickettsiales (Fig. 4a and Additional file 3). The relative abundances of the OTUs of Rickettsiales were 70.45%–94.47%, 85.66%–99.13%, and 34.45%–55.45% in the leaf, stem, and root parts, respectively (Fig. 4a and Additional file 3). Compared with that of the untreated parts, the relative abundances of bacterial Rickettsiales in the leaf part were remarkably increased by 15.38%–22.08% under drought treatment for 1, 3, and 8 days, whereas those of Rickettsiales were decreased by 6.06%–12.30% and 14.27%–41.82% in the stem and root parts, respectively (Fig. 4a and Additional file 3). The relative abundances of bacterial Rhizobiales were higher by 28.73%–97.93% in the root part treated by drought treatment for 1–8 days than that of the untreated part (Fig. 4a and Additional file 3: Dataset S6).

Moreover, the main orders of fungal communities in different parts of *C. pilosula* comprised Hypocreales, Pleosporales, Ascomycota, and Glomerellales (Fig. 4b and Additional file 3). Among these orders, the average relative abundance of Hypocreales were 18.69%, 76.95%, and 67.23% in the leaf, stem, and root parts, respectively, while those of Pleosporales were 48.50%, 3.87%, and 10.02%, respectively (Fig. 4b and Additional file 3). The relative abundances of fungal communities were remarkably different in the leaf part between the drought treatment and untreated groups (Fig. 4b and Additional file 3: Dataset S1). The relative abundances of fungal Hypocreales in the stem part subjected to drought treatment for 1, 3, and 8 days were decreased by 25.89%–28.66%, 13.48%–14.34%, and 85.35%–88.97%, respectively, relative to those in the control stem (Fig. 4b and Additional file 3: Dataset S3). The relative abundances of fungal Pleosporales in the root part under drought treatment for 1 and 8 days were lower by 29.64%–58.55% than those in untreated root part, whereas those of Hypocreales were higher by 20.87%–46.31% (Fig. 4b and Additional file 3: Dataset S5). These results revealed that the composition of endophytic community in *C. pilosula* was tissue-specific and altered by drought stress.

### Biomarkers of endophytes within *C. pilosula* related to drought stress and their correlations with the contents of secondary metabolites

**Fig. 5:**
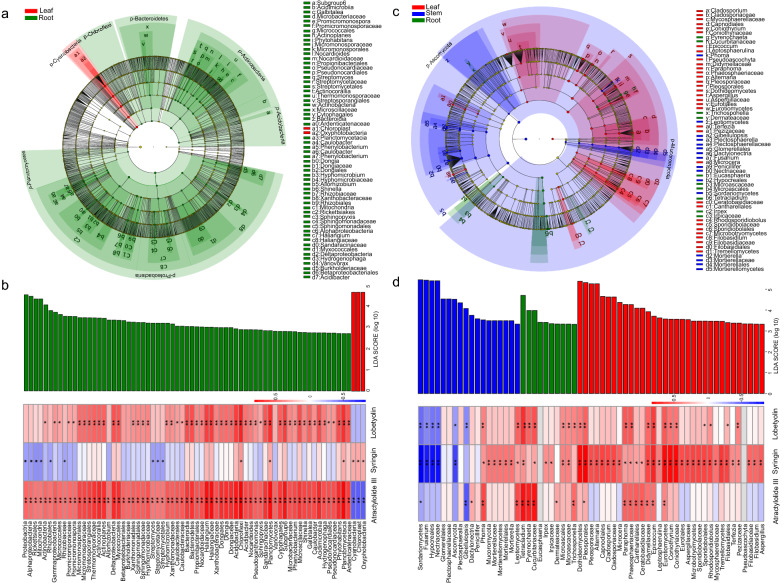
LEfSe and correlation analyses of endophytic communities in different parts of *C. pilosula.* **a**, **c** Cladogram of remarkably enriched bacterial and fungal taxa, respectively. Phylum, class, order, family, and genus levels are listed in order from the inside to the outside of the cladogram. Small circles with different colors in the diagram denote enriched taxa, while yellow circles represent the taxa with no remarkable differences in different parts. Each circle diameter is proportional to the taxon’s abundance. **b** and **d** Enriched bacterial and fungal taxa that reached a linear discriminant analysis (LDA) significance threshold of 2.5 or greater, respectively, and correlation heatmap of the relationship between the abundance of enriched bacterial and fungal taxa, respectively, and the contents of pharmacological components. Asterisks denote significant correlation based on Spearman’s analysis (**P < 0.01; *P < 0.05). *CK* untreated group, *DT* drought treatment group

LEfSe was performed to determine the microbiome members that could be used as biomarkers, and then Spearman’s correlation analysis revealed the relationships between the abundances of biomarkers and the contents of pharmacological components. 70 bacterial taxa were enriched as potential biomarkers in different parts of *C. pilosula* (leaf: 3; root: 67; Fig. 5a). These bacterial taxa belonged to seven phyla, namely Proteobacteria, Actinobacteria, Bacteroidetes, Acidobacteria, Chloroflexi, Planctomycetes, and Cyanobacteria (Fig. 5a). A total of 54, 6, and 67 biomarkers were strongly and positively associated with contents of lobetyolin, syringin, and atractylolide III, respectively; among biomarkers, most were mainly belonging to the orders of Proteobacteria, Chloroflexi, and Planctomycetes (Fig. 5b and Additional file 2: Figure S1a)*.* A total of 61 fungal taxa from Ascomycota and Mucoromycota were identified as potential biomarkers in different parts of *C. pilosula* (leaf: 33; stem: 18; root: 10; Fig. 5c). Moreover, 21, 45, and 16 biomarkers were substantially and positively correlated with the contents of lobetyolin, syringin, and atractylolide III, respectively (Fig. 5d and Additional file 2: Figure S1a). These biomarkers mainly belonged to Leotiomycetes, Tetracladium, Phaeosphaeriaceae, Cantharellales, Ceratobasidiaceae, and Didymellaceae (Fig. 5d)*.* These results suggested that the distribution of pharmacological components in the different parts of *C. pilosula* was correlated with the composition of endophytes.

**Fig. 6:**
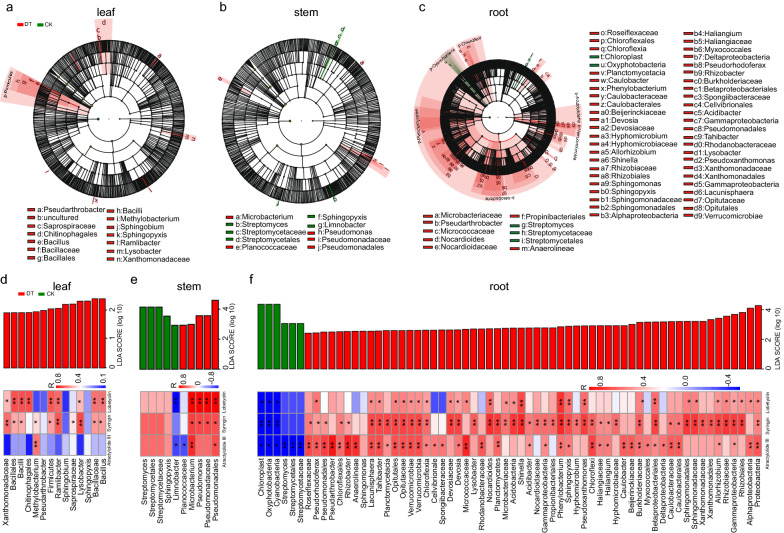
LEfSe and correlation analysis of endophytic bacteria in different parts of *C. pilosula* under drought stress. **a**–**c** Cladogram showing remarkably enriched bacterial taxa in leaf (**a**), stem (**b**), and root (**c**) parts. Phylum, class, order, family, and genus levels are listed in order from the inside to the outside of the cladogram. Small circles with different colors in the diagram denote enrichment taxa, while yellow circles represent taxa with no significant differences in different parts. Each circle diameter is proportional to the abundance of taxon. **d**–**f** Correlation heatmap of the relationship between the abundances of enriched bacterial taxa (the LDA significance threshold of 2.0 or greater) and the contents of pharmacological components in leaf (**d**), stem (**e**), and root (**f**) parts. Asterisks denote significant correlation based on Spearman’s analysis (**P < 0.01; *P < 0.05). *CK* untreated group, *DT* drought treatment group

Based on the comparison between untreated and drought treatment parts, 14, 10, and 64 bacterial taxa were enriched as potential biomarkers in the leaf, stem, and root parts, respectively (Fig. 6a-c). These potential biomarkers mainly belonged to Firmicutes, Cyanobacteria, Verrucomicrobia, Planctomycetes, Acidobacteria, Chloroflexi, and Proteobacteria (Fig. 6a–c). Compared with those in the control *C. pilosula*, the abundances of 11, 5, and 58 potential biomarkers in the leaf, stem, and root parts under drought treatment were increased by 20.00%–95.24%, 52.27%–84.91%, and 10.96%–78.03%, respectively (Additional file 2: Figure S1b–S1d and Additional file 3). The abundances of 9, 10, and 1 bacterial biomarkers in the leaf part were positively associated with the contents of lobetyolin, syringin, and atractylolide III, respectively (Fig. 6d). The abundances of 4, 4, and 2 bacterial biomarkers in the stem part were positively correlated with the contents of lobetyolin, syringin, and atractylolide III, respectively (Fig. 6e). The abundances of 28, 46, and 40 bacterial biomarkers in the root part were positively related to the contents of lobetyolin, syringin, and atractylolide III, respectively (Fig. 6f). These biomarkers mainly belonged to Xanthomonadaceae, Bacillales, *Methylobacterium*, and *Ramlibacter* in the leaf part (Fig. 6d); *Microbacterium* and Pseudomonadales in the stem part (Fig. 6e); and Pseudorhodoferax, *Lacunisphaera*, *Tahibacter*, Verrucomicrobiae, *Verrucomicrobia*, and Chloroflexia in the root part (Fig. 6f).

**Fig. 7:**
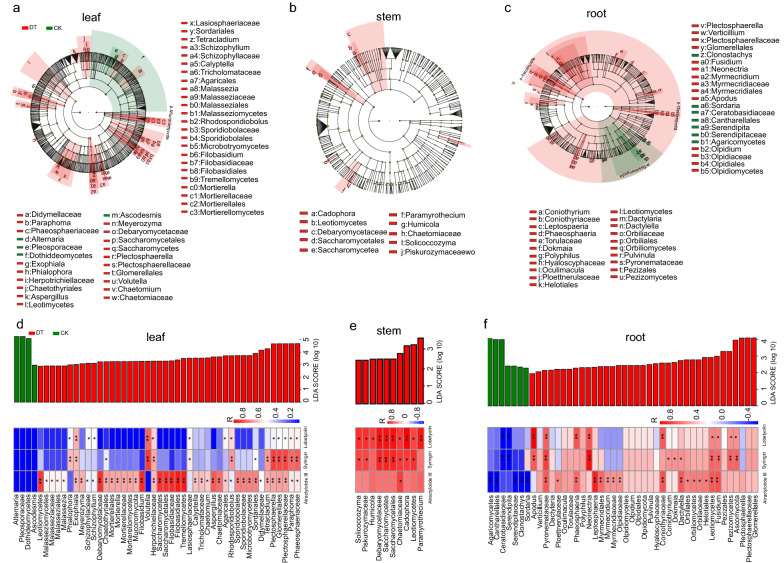
LEfSe and correlation analysis of endophytic fungi in different parts of *C. pilosula* under drought stress. **a**–**c** Cladogram showing remarkably enriched fungal taxa in leaf (**a**), stem (**b**), and root (**c**) parts. Phylum, class, order, family, and genus levels are listed in order from the inside to the outside of the cladogram. Small circles with different colors in the diagram denote enrichment taxa, while yellow circles represent taxa with no significant differences in different parts. Each circle diameter is proportional to the abundance of taxon. **d**–**f** Correlation heatmap of the relationship between the abundances of enriched fungal taxa (the LDA significance threshold of 2.0 or greater) and the contents of pharmacological components in leaf (**d**), stem (**e**), and root (**f**) parts. Asterisks denote significant correlation based on Spearman’s analysis (**P < 0.01; *P < 0.05). *CK* untreated group, *DT* drought treatment group

In accordance with the comparison of the untreated and drought treatment, 48, 10, and 44 fungal taxa were identified as potential biomarkers in the leaf, stem, and root parts, respectively (Fig. 7a–c). The potential biomarkers belonged to Basidiomycota, Mucoromycota, Olpidiomycota, and Ascomycota (Fig. 7a–c). In comparison with those in untreated parts of *C. pilosula*, the abundances of 44, 10, and 38 potential biomarkers in the leaf, stem, and root parts after drought treatment increased by over 121.00%, 824.00%, and 33.60%, respectively (Additional file 2: Figure S1b–S1d and Additional file 3). The abundances of 14, 14, and 37 fungal biomarkers in the leaf part were positively correlated with the contents of lobetyolin, syringin, and atractylolide III, respectively (Fig. 7d). The abundances of 10, 1, and 8 fungal biomarkers in stem part were positively associated with the contents of lobetyolin, atractylolide III, and syringin, respectively (Fig. 7e). The abundances of 9, 10, and 16 fungal biomarkers in the root part were positively related to the contents of lobetyolin, syringin, and atractylolide III, respectively (Fig. 7f). These biomarkers mainly belonged to Exophiala, Herpotrichiellaceae, *Plectosphaerella*, Glomerellales, Plectosphaerellceae, *Paraphoma*, and Phaeosphaeriaceae in the leaf part (Fig. 7d); Chaetomiaceae, *Solicoccozyma*, and Piskurozymaceae in the stem part (Fig. 7e); and Pyronemataceae, Leotiomycetes, and *Fusidium* in the root part (Fig. 7f). These results suggested that the endophytes were related to the contents of pharmacological components and might be involved in the accumulations of pharmacological components in *C. pilosula* parts under drought stress. In the following experiments, we further verified the function of endophytes in metabolite accumulations.

### Endophytes participated in the accumulations of pharmacodynamic compounds

**Fig. 8:**
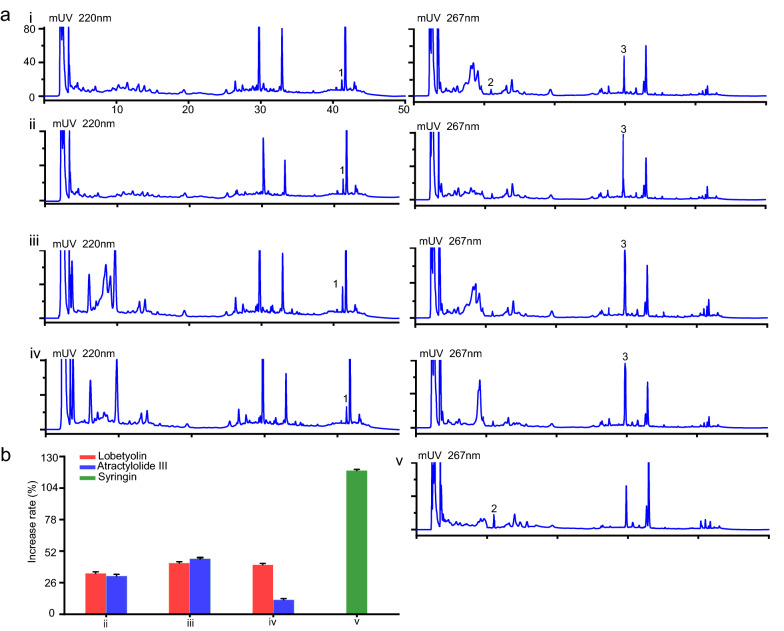
Increase rates of the lobetyolin, syringin, and atractylolide III contents in *C. pilosula* grafted with endophytes. **a** The HPLC chromatograms of samples. **b** Increase rates of the lobetyolin, syringin, and atractylolide III contents. Columns represent the means of three repeats ± standard deviation (SD). The red, blue, and green colors represent the lobetyolin, atractylolide III, and syringin, respectively. 1. atractylolide III; 2. syringin; 3. lobetyolin. i, *C. pilosula* powder + medium control. ii, *C. pilosula* powder + fungus *Epicoccum thailandicum*. iii, *C. pilosula* powder + fungus *Filobasidium magnum*. iv, *C. pilosula* powder + fungus *Paraphoma rhaphiolepidis*. v, *C. pilosula* powder + bacterium *Pseudomonas nitroreducens*

Three endophytic bacteria and seven endophytic fungi obtained from the microbiological culture collection library of the laboratory were cultured with sterile *C. pilosula* powder to verify the functions of endophytes in metabolite accumulations. Following incubation, the bacterium *Pseudomonas nitroreducens* and the fungi *Epicoccum thailandicum*, *Filobasidium magnum*, and *Paraphoma rhaphiolepidis* were confirmed to be involved in the accumulations of pharmacodynamic compounds (Fig. 8a, b). Lobetyolin, syringin, and atractylolide III were not detected in groups (endophyte + medium). The contents of lobetyolin and atractylolide III was increased by *E. thailandicum* at the rates of 33.14‒%34.70% and 30.24%‒31.40%, respectively; by *F. magnum* at the rates of 41.89%‒42.48% and 45.7%‒46.02%, respectively; and by *P. rhaphiolepidis* at the rates of 39.45%‒40.93% and 11.12%‒11.98%, respectively (Fig. 8b). And *P. nitroreducens* could increase syringin content at the rates of 118.61%‒119.36% (Fig. 8b).

## Discussion

In this study, the distribution of the pharmacological components of *C. pilosula* had tissue specificity, which was similar to the observations in the study of *Panax quinquefolius* and *Panax notoginseng* [33]. This work provided references for rational use of the different *C. pilosula* parts. The contents of lobetyolin, syringin, and atractylolide III in drought treatment parts of *C. pilosula* were higher than those in untreated parts. This result was supported by another report on *C. pilosula*, where the content of lobetyolin in *C. pilosula* parts under drought treatment was also remarkably increased compared with those in control parts [9]. These highly accumulated pharmacological components in *C. pilosula* could provide protections against damages of drought stress [33–35]. For example, Syringin, atractylolide, α–tocopherol, flavonol, and anthocyanin as non–enzymatic antioxidants could scavenge ROS to protect cell proteins, enzymes, organelles, cell membranes and maintain normal plant growth [36, 37]; Organic acids could regulate the osmotic pressure of plant cell, thus prevented excessive water loss, held cell turgor, and kept the physiological processes of plants [38, 39]. The results indicated that moderate drought stress was conducive to the accumulation of secondary metabolites for facilitating the adaptation of medicinal plants to drought stress.

We would elucidate the underlying mechanism of the accumulations of secondary metabolites in *C*. *pilosula* parts under drought stress. The endophytes, an important member of the ecosystem, had irreplaceable functions in promoting growth of medicinal plants [10]. In this work, the diversity and composition of endophytes also showed tissue-dependent in *C. pilosula*, and these results were consistent with those in *Panax notoginseng* [40]. The diversity of endophytic communities was higher in *C. pilosula* parts with drought treatment than those in untreated parts, similar results were reported in the endophytic bacteria of *Leymus chinensis* under drought stress [41, 42]. The composition of endophytes in *C. pilosula* parts under water deficit was markedly different from that in control parts, which was in agreement with the findings in *Sorghastrum nutans* [43]. PCoA also showed that endophytic communities from *C. pilosula* parts with drought treatment were distinguishable from those in untreated parts. The endophytic bacteria in wheat under drought stress were definitely different from those in control samples [44]. Previous studies showed that the increase in endophytic diversity and the variation in composition were beneficial to the formation of complexes and stable networks, which might enhance the endophyte, endophyte–endophyte, and endophyte–plant interaction to implement the potential functions of the microorganism [41]. Besides, the diversity and composition of endophytes were reset under drought stress to enrich specific beneficial microorganisms in response to adversity [45, 46]. The results indicated that endophytes in *C. pilosula* parts were sensitive to drought and could quickly respond to drought stress. Further analysis, LEfSe was used to determine the microbiome members as biomarkers, and then Spearman’s correlation showed that the abundances of biomarkers, such as *Plectosphaerella*, *Microbacterium*, Chaetomiaceae, Pseudomonadales, *Allorhizobium*, and Chloroflexia, were positively related to the contents of pharmacodynamic compounds in *C. pilosula* parts under drought treatment. A significant and positive correlation existed between *Bacillus* abundance and syringin content, this finding was consistent with other studies on *Magnolia ofcinalis* Rehd. et Wils [47]. *Pestalotiopsis neglecta* BAB–5510 was found to be related to the accumulation of phenols, flavonoids, terpenoids, alkaloids, tannins, carbohydrates, and saponin in *Cupressus torulosa* [48]. Many endophytes, such as Burkholderiaceae, *Allorhizobium*, and *Pseudomonas*, were also beneficial to the productions of ginsenoside, sesquiterpenoid, and alkaloid in medicinal plants [49, 50]. These evidences indicated that the endophytes were associated with the contents of pharmacodynamic compounds in *C. pilosula* parts under drought stress and might be involved in the productions of pharmacological components.

The data of culture-dependent experiments showed that contents of lobetyolin and atractylolide III were increased by *E. thailandicum*, *F. magnum* and *P. rhaphiolepidis*, and *P. nitroreducens* increased syringin content. *Glomus moseae*, an endophytic fungus, could stimulate *Glycyrrhiza uralensis* to accumulate soluble sugar and proline to reduce water potential of cell and hold osmotic pressure under drought stress [51]. *Bacillus licheniformis*, an endophyte of *Pinellia ternata*, could generate non–enzymatic antioxidants (alkaloids) for maintaining the homeostasis of reactive oxygen species in plant [52]. The mechanisms of endophytes on the accumulation of metabolites in the host mainly included induction and transformation [53]. Endophytes produced biochemical signal molecules, such as glycoprotein, chitosan, chitin, cyclodextrin, small peptide segment, and unsaturated fatty acid, to rapidly induce the expression of specific genes for activation of a series of secondary metabolic pathways, thus promoting accumulations of active compounds in medicinal plants [45]. Besides, endophytes synthesized key enzymes to transform the precursor ingredients into active components in medicinal plants [53]. The endophytes of *Camptotheca acuminata*, such as *Fusarium solani*, *Phomopsis* sp., *Alternaria* sp., and *Colletotrichum* sp., synthesized key enzymes lacking in plant, and these enzymes converted the precursors produced by *C. acuminata* into camptothecin and its analogues [51]. The results of the present study further demonstrated that microbial biomarkers participated in accumulation of the secondary metabolites, and they played synergetic roles in increasing the contents of pharmacodynamic compounds in *C. pilosula* under drought stress.

## Conclusions

In summary, the diversity and composition of endophytes and the distribution of pharmacological components showed tissue specificity in *C. pilosula*. Drought stress contributed to the accumulation of pharmacological components and the increase of endophytic diversity in *C. pilosula.* Correlation analysis revealed that the accumulation of pharmacological components in *C. pilosula* were related to the dynamic changes of endophytes. And further culture-dependent experiments validated that endophytes participated in the accumulation of metabolites. This study laid a solid foundation for the exploitation of endophytes to enhance curative effect and utilization value of Codonopsis Radix.

## Supplementary Information

**Additional file 1. **Validation for HPLC–UV method.

**Additional file 2: Figure S1.** Abundances of enriched taxa in different parts of *C. pilosula*. **Table S1.** Numbers and average lengths of microbial sequences in leaf part of *C. pilosula*. **Table S2.** Numbers and average lengths of microbial sequences in stem part of *C. pilosula*. **Table S3.** Numbers and average lengths of microbial sequences in root part of *C. pilosula*.

**Additional file 3: **OTU abundance of endophytes in different parts of *C. pilosula*.

## Data Availability

The 16S rRNA and ITS rRNA gene raw sequence data of leaf, stem, and root parts were submitted to the NCBI SAR database (http://www.ncbi.nlm.nih.gov/) under accession numbers PRJNA721564, PRJNA721933, and PRJNA663111, respectively.

## Abbreviations

ROS: Reactive oxygen species
HPLC: High–performance liquid chromatography
CK: Untreated group
DT: Drought treatment group
PCoA: Principal coordinate analysis
SD: Standard deviation
NCBI: National Center for Biotechnology Information
OTUs: Operational taxonomic units
LEfSe: Linear discriminant effect size
LDA: Linear discriminant analysis

## References

[1] Kim YJ, Zhang D, Yang DC (2015). Biosynthesis and biotechnological production of ginsenosides. Biotechnol Adv.

[2] Bai RB, Zhang YJ, Fan JM, Jia XS, Li D, Wang YP (2020). Immune-enhancement effects of oligosaccharides from *Codonopsis pilosula* on cyclophosphamide induced immunosuppression in mice. Food Funct.

[3] He JY, Ma N, Zhu S, Komatsu K, Li ZY, Fu WM (2015). The genus *Codonopsis* (Campanulaceae): a review of phytochemistry, bioactivity and quality control. J Nat Med.

[4] He JY, Zhu S, Goda Y, Cai SQ, Komatsu K (2014). Quality evaluation of medicinally-used *Codonopsis* species and Codonopsis Radix based on the contents of pyrrolidine alkaloids, phenylpropanoid and polyacetylenes. J Nat Med.

[5] Gao S, Liu J, Wang M, Liu Y, Meng X, Zhang T (2019). Exploring on the bioactive markers of Codonopsis Radix by correlation analysis between chemical constituents and pharmacological effects. J Ethnopharmacol.

[6] Tsai KH, Lee NH, Chen GY, Hu WS, Tsai CY, Chang MH (2013). Dung-shen (*Codonopsis pilosula*) attenuated the cardiac-impaired insulin-like growth factor II receptor pathway on myocardial cells. Food Chem.

[7] Lau KM, Yue GG, Chan YY, Kwok HF, Gao S, Wong CW (2019). A review on the immunomodulatory activity of *Acanthopanax senticosus* and its active components. Chin Med.

[8] Niu HS, Liu IM, Cheng JT, Lin CL, Hsu FL (2008). Hypoglycemic effect of syringin from *Eleutherococcus senticosus* in streptozotocin-induced diabetic rats. Planta Med.

[9] Lin LC, Tsai TH, Kuo CL (2013). Chemical constituents comparison of *Codonopsis tangshen*, *Codonopsis pilosula* var. modesta and *Codonopsis pilosula*. Nat Prod Res..

[10] Nagabhyru P, Dinkins RD, Wood CL, Bacon CW, Schardl CL (2013). Tall fescue endophyte effects on tolerance to water-deficit stress. BMC Plant Biol.

[11] Liarzi O, Bucki P, Braun Miyara S, Ezra D (2016). Bioactive volatiles from an endophytic Daldinia cf. concentrica isolate affect the viability of the plant parasitic nematode Meloidogyne javanica. PLoS ONE..

[12] Liu Y, Liu W, Liang Z (2015). Endophytic bacteria from *Pinellia ternata*, a new source of purine alkaloids and bacterial manure. Pharm Biol.

[13] Gao Y, Lu Q, Zang P, Li X, Ji Q, He ZM (2015). An endophytic bacterium isolated from *Panax ginseng* CA Meyer enhances growth, reduces morbidity, and stimulates ginsenoside biosynthesis. Phytochem Lett.

[14] van de Veerdonk FL, Gresnigt MS, Romani L, Netea MG, Latgé JP (2017). *Aspergillus fumigatus* morphology and dynamic host interactions. Nat Rev Microbiol.

[15] Chen F, Ren CG, Zhou T, Wei YJ, Dai CC (2016). A novel exopolysaccharide elicitor from endophytic fungus *Gilmaniella* sp. AL12 on volatile oils accumulation in *Atractylodes lancea*. Sci Rep..

[16] Zhou JY, Yuan J, Li X, Ning YF, Dai CC (2015). Endophytic bacterium triggered reactive oxygen species directly increase oxygenous sesquiterpenoid content and diversity in *Atractylodes lancea*. Appl Environ Microbiol.

[17] Liang Y, Wei G, Ning K, Li M, Zhang G, Luo L (2021). Increase in carbohydrate content and variation in microbiome are related to the drought tolerance of *Codonopsis pilosula*. Plant Physiol Biochem.

[18] May JC, Wheeler RM, Grim E (1989). The gravimetric method for the determination of residual moisture in freeze-dried biological products. Cryobiology.

[19] Marthandan V, Geetha R, Kumutha K, Renganathan VG, Karthikeyan A, Ramalingam J (2020). Seed priming: a feasible strategy to enhance drought tolerance in crop plants. Int J Mol Sci.

[20] Ruiz-Nieto JE, Aguirre-Mancilla CL, Acosta-Gallegos JA, Raya-Pérez JC, Piedra-Ibarra E, Vázquez-Medrano J (2015). Photosynthesis and chloroplast genes are involved in water-use efficiency in common bean. Plant Physiol Biochem.

[21] Dong LL, Xu J, Li Y, Fang HL, Niu WH, Li XW (2018). Manipulation of microbial community in the rhizosphere alleviates the replanting issues in *Panax ginseng*. Soil Biol Biochem.

[22] Zhang Q, Wang L, Liu Z, Zhao Z, Zhao J, Wang Z (2020). Transcriptome and metabolome profiling unveil the mechanisms of *Ziziphus jujuba* Mill peel coloration. Food Chem..

[23] Dong L, Xu J, Zhang L, Cheng R, Wei G, Su H, Yang J (2018). Rhizospheric microbial communities are driven by *Panax ginseng* at different growth stages and biocontrol bacteria alleviates replanting mortality. Acta Pharm Sin B.

[24] Jiao S, Xu Y, Zhang J, Hao X, Lu Y (2019). Core microbiota in agricultural soils and their potential associations with nutrient cycling. mSystems..

[25] Mueller RC, Paula FS, Mirza BS, Rodrigues JL, Nüsslein K, Bohannan BJ (2014). Links between plant and fungal communities across a deforestation chronosequence in the Amazon rainforest. ISME J.

[26] Fierer N, Hamady M, Lauber CL, Knight R (2008). The influence of sex, handedness, and washing on the diversity of hand surface bacteria. Proc Natl Acad Sci USA.

[27] Magoč T, Salzberg SL (2011). FLASH: fast length adjustment of short reads to improve genome assemblies. Bioinformatics.

[28] Kong Y (2011). Btrim: a fast, lightweight adapter and quality trimming program for next-generation sequencing technologies. Genomics.

[29] Lozupone C, Knight R (2005). UniFrac: a new phylogenetic method for comparing microbial communities. Appl Environ Microbiol.

[30] Segata N, Izard J, Waldron L, Gevers D, Miropolsky L, Garrett WS (2011). Metagenomic biomarker discovery and explanation. Genome Biol.

[31] Wei G, Chen Z, Wang B, Wei F, Zhang G, Wang Y (2021). Endophytes isolated from *Panax notoginseng* converted ginsenosides. Microb Biotechnol.

[32] Fu Y, Yin ZH, Yin CY (2017). Biotransformation of ginsenoside Rb1 to ginsenoside Rg3 by endophytic bacterium *Burkholderia* sp. GE 17–7 isolated from Panax ginseng. J Appl Microbiol..

[33] Wei G, Yang F, Wei F, Zhang L, Gao Y, Qian J (2020). Metabolomes and transcriptomes revealed the saponin distribution in root tissues of *Panax quinquefolius* and *Panax notoginseng*. J Ginseng Res.

[34] Lu H, Hu Y, Wang C, Liu W, Ma G, Han Q, Ma D (2019). Effects of high temperature and drought stress on the expression of gene encoding enzymes and the activity of key enzymes involved in starch biosynthesis in wheat grains. Front Plant Sci.

[35] Naderi S, Fakheri BA, Maali-Amiri R, Mahdinezhad N (2020). Tolerance responses in wheat landrace Bolani are related to enhanced metabolic adjustments under drought stress. Plant Physiol Biochem.

[36] Nakabayashi R, Mori T, Saito K (2014). Alternation of flavonoid accumulation under drought stress in Arabidopsis thaliana. Plant Signal Behav..

[37] Mittler R (2002). Oxidative stress, antioxidants and stress tolerance. Trends Plant Sci.

[38] Szabados L, Savouré A (2010). Proline: a multifunctional amino acid. Trends Plant Sci.

[39] Isah T (2019). Stress and defense responses in plant secondary metabolites production. Biol Res.

[40] Dong L, Cheng R, Xiao L, Wei F, Wei G, Xu J (2018). Diversity and composition of bacterial endophytes among plant parts of *Panax notoginseng*. Chin Med.

[41] Zhou H, Gao Y, Jia XH, Wang MM, Ding JJ, Cheng L (2020). Network analysis reveals the strengthening of microbial interaction in biological soil crust development in the Mu Us Sandy Land, northwestern China. Soil Biol Biochem..

[42] Li J, Meng B, Chai H, Yang X, Song W, Li S (2019). Arbuscular Mycorrhizal Fungi Alleviate Drought Stress in C_3_ (*Leymus chinensis*) and C_4_ (*Hemarthria altissima*) Grasses via Altering Antioxidant Enzyme Activities and Photosynthesis. Front Plant Sci.

[43] Naylor D, DeGraaf S, Purdom E, Coleman-Derr D (2017). Drought and host selection influence bacterial community dynamics in the grass root microbiome. ISME J.

[44] Žiarovská J, Medo J, Kyseľ M, Zamiešková L, Kačániová M (2020). Endophytic bacterial microbiome diversity in early developmental stage plant tissues of wheat varieties. Plants.

[45] Zhai X, Jia M, Chen L, Zheng CJ, Rahman K, Han T (2017). The regulatory mechanism of fungal elicitor-induced secondary metabolite biosynthesis in medical plants. Crit Rev Microbiol.

[46] Hu W, Zhang H, Chen H, Tang M (2017). Arbuscular mycorrhizas influence *Lycium barbarum* tolerance of water stress in a hot environment. Mycorrhiza.

[47] Wu Q, Wei D, Dong L, Liu Y, Ren C, Liu Q (2019). Variation in the microbial community contributes to the improvement of the main active compounds of Magnolia officinalis Rehd. et Wils in the process of sweating. Chin Med..

[48] Wang J, Peng Q, Yao X, Liu Y, Zhou X (2020). New pestallic acids and diphenylketone derivatives from the marine alga-derived endophytic fungus *Pestalotiopsis neglecta* SCSIO41403. J Antibiot.

[49] Hardoim PR, Overbeek LS, Elsas JD (2008). Properties of bacterial endophytes and their proposed role in plant growth. Trends Microbiol.

[50] Dong L, Li Y, Xu J, Yang J, Wei G, Shen L (2019). Biofertilizers regulate the soil microbial community and enhance *Panax ginseng* yields. Chin Med.

[51] Kusari S, Hertweck C, Spiteller M (2012). Chemical ecology of endophytic fungi: origins of secondary metabolites. Chem Biol.

[52] Kusari S, Zühlke S, Spiteller M (2009). An endophytic fungus from Camptotheca acuminata that produces camptothecin and analogues. J Nat Prod.

[53] Liu G, Lai D, Liu QZ, Zhou L, Liu ZL (2016). Identification of nematicidal constituents of *Notopterygium incisum* Rhizomes against *Bursaphelenchus xylophilus* and *Meloidogyne incognita*. Molecules.

