# Supplementary material for: Contents of lobetyolin, syringin, and atractylolide III in Codonopsis pilosula are related to dynamic changes of endophytes under drought stress
Source: Chin Med. 2021 Nov 22;16:122. doi: 10.1186/s13020-021-00533-z (PMC8607676; doi:10.1186/s13020-021-00533-z)
Supplement: Supplementary file 1 — Additional file 1. Validation for HPLC–UV method. [file 13020_2021_533_MOESM1_ESM.doc]

**Method validation**

**1. Materials and Methods**

**1.1 Chemicals, Standards and Apparatus**

Methanol, acetonitrile, and phosphoric acid were of chromatographic grade, and purchased from Fisher Controls International, LLC (Iowa, USA). Lobetyolin, syringin, and atractylolide III standards (purity ≥ 98.0%; batch numbers: CHB180224, CHB180530, and E1708006, respectively) were purchased from Chengdu Croma Biotechnology Co., Ltd (Chengdu, China).

The analytical balance ME203E with a precision of 0.1 mg (Mettler Toledo, Switzerland); the centrifuge 5425R (Eppendorf, Germany); the ultrasonic water bath DK–8D (Senxin, China); and the Agilent HPLC–UV 1260 series system (Agilent, USA) equipped with a quaternary pump, an automatic sampler, a column compartment, and a variable wavelength detector were used in this study. The chromatogram column was a C_18_ column (4.6 mm × 250 mm, 5 µm; Eclipse XDB; Agilent, USA).

**1.2 HPLC Conditions**

For HPLC analysis, the flow rate was 1.0 mL·min^−1^, the column temperature was 25 °C, the injection volume was 20 μL, and UV detection was performed at 267 (lobetyolin and syringin) and 220 nm (atractylolide III). The mobile phase consisted of acetonitrile (A) and water containing 0.1% phosphoric acid (v/v) (B). The gradient elution conditions were set as follows: 0–10 min, 5%–15% A; 10–20 min, 15% A; 20–35 min, 15%–45% A; 35–40 min, 45%–85% A; 40–45 min, 85%–5% A; and 45–50 min, 5% A [1].

**1.3 Sample Preparation**

Approximately 0.1 g of powder was extracted with 1.5 mL methanol under ultrasound for 40 min. After centrifugation at 4,000 × g for 10 min, the supernatant was passed through a 0.22 μm microporous membrane [1].

**1.4 Preparation of Standard Solution**

Individual stock solutions of the three standard compounds were prepared in methanol solvent at a concentration of 1.0 mg·mL^−1^, and then stock solutions were diluted with methanol to obtain standard solutions of different concentrations (lobetyolin: 60.12, 137.78, 217.33, 300.25, 378.81, 450.73, 521.90, and 604.56 μg·mL^−1^; syringin: 0.71, 4.85, 9.03, 12.92, 17.32, 21.56, 25.89, and 29.43 μg·mL^−1^; atractylolide III: 0.52, 9.74, 18.02, 26.41, 34.66, 42.96, 51.28, and 60.61 μg·mL^−1^).

**1.5 Validation**

Precision and repeatability: intraday and interday precisions were evaluated by replicate injections of standard and sample solutions. Five injections per day were conducted for 3 days [2].

Limit of detection (LOD) and limit of quantitation (LOQ): the standard solutions of the low concentration were further diluted with methanol to determine LOD and LOQ at the signal–to–noise ratios (s/n) of 3:1 and 10:1, respectively [3].

Linearity: more than six–point calibration curves of each standards showed a linear correlation between the concentration of standards and peak area.

Recovery: Three standards of approximately 50%, 100%, and 150% of the original amounts in the samples were added to the samples, and then they were extracted and analyzed at three replicates per sample.

**2. Results**

The method had good peak separation and presented good intraday and interday precision (RSD < 3.28%; Fig. S1 and Table 1). The linear ranges of lobetyolin, syringin, and atractylolide III were 60.12–521.90, 0.71–25.89, and 0.52–60.61 μg·mL^−1^, respectively, and their correlation coefficients (*r*) were greater than 0.9989 (Table 2). LOD and LOQ were 0.12–0.27 and 0.38–0.83 μg·mL^−1^, respectively (Table 2). The recoveries of the added standards ranged from 95.5 to 103.7%, with an RSD < 4.01% (Table 3). The validation experiment indicated that the method was suitable for routine analysis of target compounds.

**
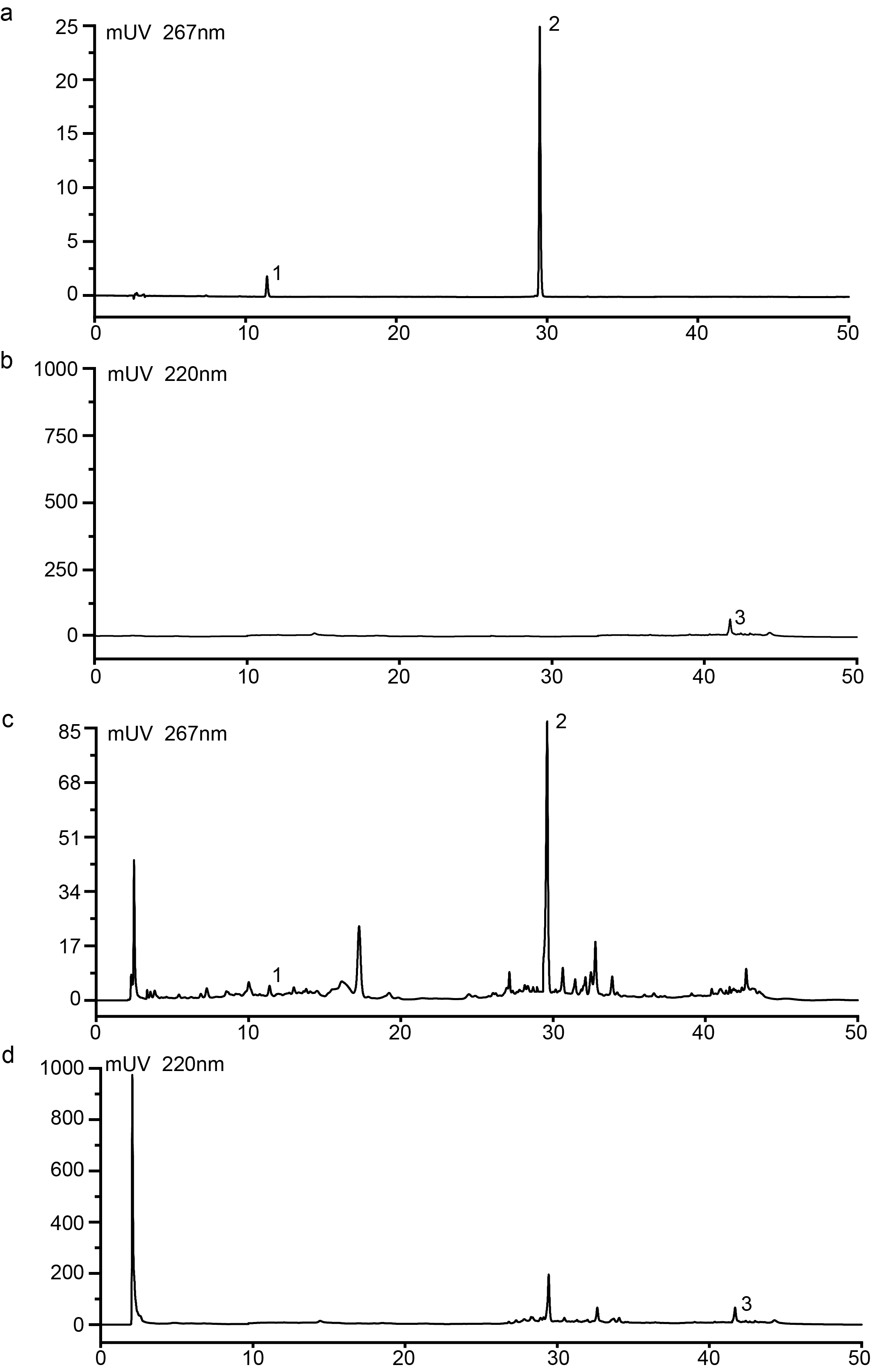
**

**Fig. S1** The HPLC chromatograms of standards and samples

(a-b) Standards. 1. syringin; 2. lobetyolin; 3. atractylolide III. (c-d) samples

**Table 1.** Intraday and interday precisions of standard and sample solutions

|  | Standard solution | | Sample solution | |
| --- | --- | --- | --- | --- |
| Compound | Intraday (RSD) | Interday (RSD) | Intraday (RSD) | Interday (RSD) |
| Lobetyolin | 1.45 | 2.56 | 2.63 | 3.28 |
| Syringin | 2.67 | 2.89 | 2.83 | 3.06 |
| Atractylolide III | 1.37 | 1.19 | 1.37 | 2.19 |

**Table 2.** Linear regression, LOD and LOQ of standard compounds

| Compound | Regression equation^a^ | Correlation coefficient (*r*) | Linear range (μg·mL^-1^) | LOD (μg·mL^-1^) | LOQ (μg·mL^-1^) |
| --- | --- | --- | --- | --- | --- |
| Lobetyolin | *y*=1008*x*+2.2498 | 1.0000 | 60.12–521.90 | 0.12 | 0.38 |
| Syringin | *y*=544.6*x*+5.2678 | 0.9989 | 0.71–25.89 | 0.25 | 0.71 |
| Atractylolide III | *y*=2252.2*x*-0.235 | 0.9999 | 0.52–60.61 | 0.27 | 0.83 |

^a^ *x* is the concentration of each compound (μg·mL^-1^); *y* is the peak area of the respective compound.

**Table 3. Recoveries of compounds**

| Compound | Original (μg) | Added (μg) | Determinated (μg) | Recovery (%) | RSD (%) |
| --- | --- | --- | --- | --- | --- |
| Lobetyolin | 114.1 | 58.2 | 174.3 | 103.4 | 3.54 |
|  |  |  | 171.5 | 98.6 |  |
|  |  |  | 170.3 | 96.6 |  |
|  |  | 112.4 | 225 | 98.7 | 1.45 |
|  |  |  | 224.8 | 98.5 |  |
|  |  |  | 227.7 | 101.1 |  |
|  |  | 168.7 | 284.9 | 101.2 | 1.35 |
|  |  |  | 281.5 | 99.2 |  |
|  |  |  | 280.6 | 98.7 |  |
| Syringin | 10.5 | 6.7 | 17.4 | 103.0 | 4.01 |
|  |  |  | 17.0 | 97.0 |  |
|  |  |  | 16.9 | 95.5 |  |
|  |  | 11.3 | 21.5 | 97.3 | 2.76 |
|  |  |  | 21.3 | 95.6 |  |
|  |  |  | 21.9 | 100.9 |  |
|  |  | 16.4 | 27.3 | 102.4 | 2.50 |
|  |  |  | 26.7 | 98.8 |  |
|  |  |  | 27.5 | 103.7 |  |
| Atractylolide III | 25.3 | 14.5 | 39.6 | 98.6 | 2.13 |
|  |  |  | 40.0 | 102.1 |  |
|  |  |  | 39.4 | 97.2 |  |
|  |  | 26.9 | 52.3 | 100.4 | 1.30 |
|  |  |  | 52.6 | 101.5 |  |
|  |  |  | 51.9 | 98.9 |  |
|  |  | 37.1 | 62.5 | 100.3 | 0.82 |
|  |  |  | 62.7 | 101.3 |  |
|  |  |  | 62.3 | 99.7 |  |

**Reference:**

[1]Lin LC, Tsai TH, Kuo CL. Chemical constituents comparison of *Codonopsis tangshen*, *Codonopsis pilosula* var. modesta and *Codonopsis pilosula*. Nat Prod Res. 2013;27(19):1812-15.

[2]He JY, Zhu S, Komatsu K. HPLC/UV analysis of polyacetylenes, phenylpropanoid and pyrrolidine alkaloids in medicinally used *Codonopsis* species. Phytochem Anal. 2014;25(3):213-9.

[3]Wang YH, Avonto C, Avula B, Wang M, Rua D, Khan IA. Quantitative Determination of α-Arbutin, β-Arbutin, Kojic Acid, Nicotinamide, Hydroquinone, Resorcinol, 4-Methoxyphenol, 4-Ethoxyphenol, and Ascorbic Acid from Skin Whitening Products by HPLC-UV. J AOAC Int. 2015;98(1):5-12.
