# Supplementary material for: Contents of lobetyolin, syringin, and atractylolide III in Codonopsis pilosula are related to dynamic changes of endophytes under drought stress
Source: Chin Med. 2021 Nov 22;16:122. doi: 10.1186/s13020-021-00533-z (PMC8607676; doi:10.1186/s13020-021-00533-z)
Supplement: Supplementary file 2 — Additional file 2: Figure S1. Abundances of enriched taxa in different parts of C. pilosula. Table S1. Numbers and average lengths of microbial sequences in leaf part of C. pilosula. Table S2. Numbers and average lengths of microbial sequences in stem part of C. pilosula. Table S3. Numbers and average lengths of microbial sequences in root part of C. pilosula. [file 13020_2021_533_MOESM2_ESM.doc]

The following supplementary data is available for this article:

**Fig. S1** Abundances of enriched taxa in different parts of *C. pilosula*.

**Table S1** Numbers and average lengths of microbial sequences in leaf part of *C. pilosula*.

**Table S2** Numbers and average lengths of microbial sequences in stem part of *C. pilosula*.

**Table S3** Numbers and average lengths of microbial sequences in root part of *C. pilosula*.

**
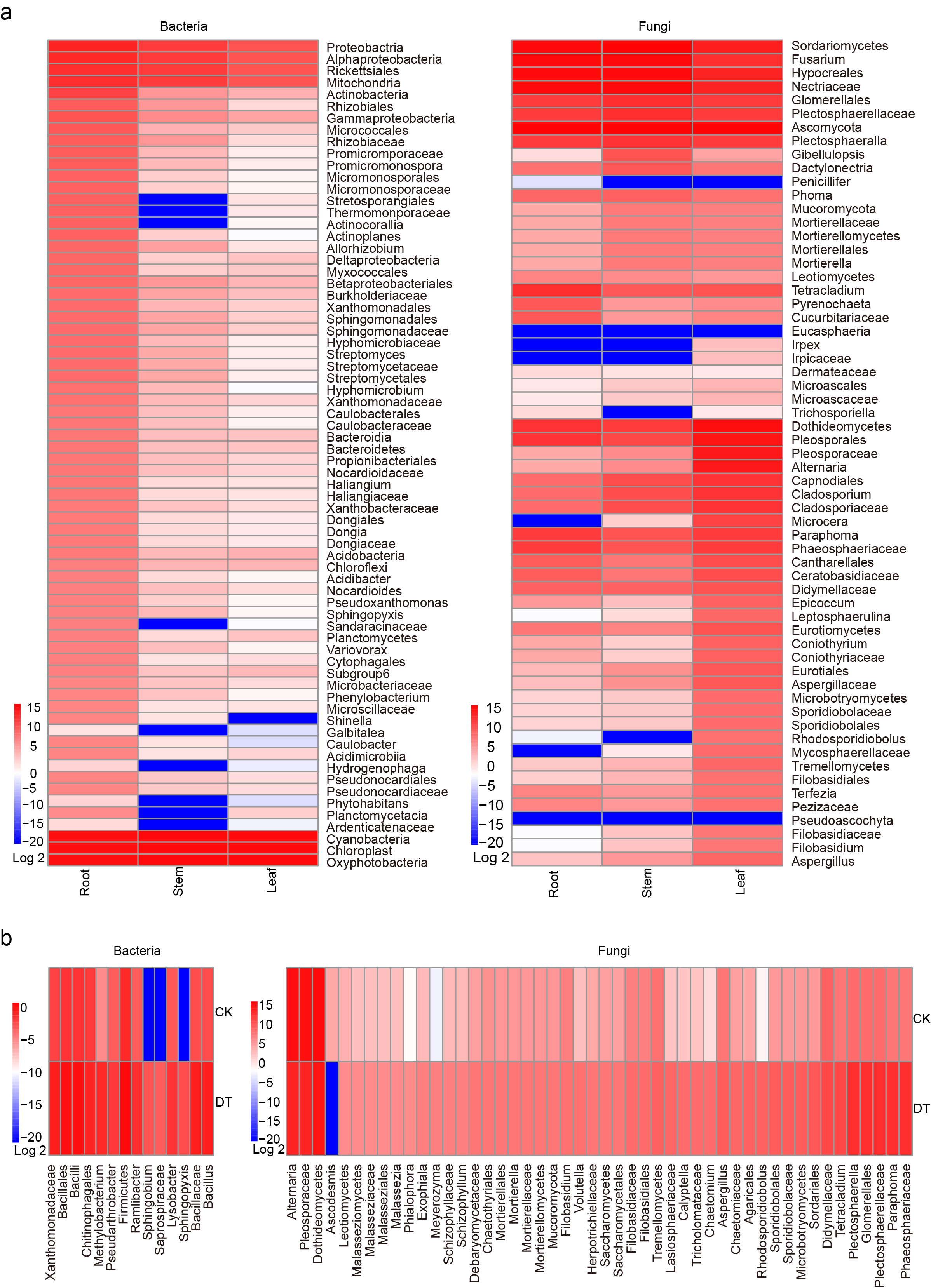
**

**
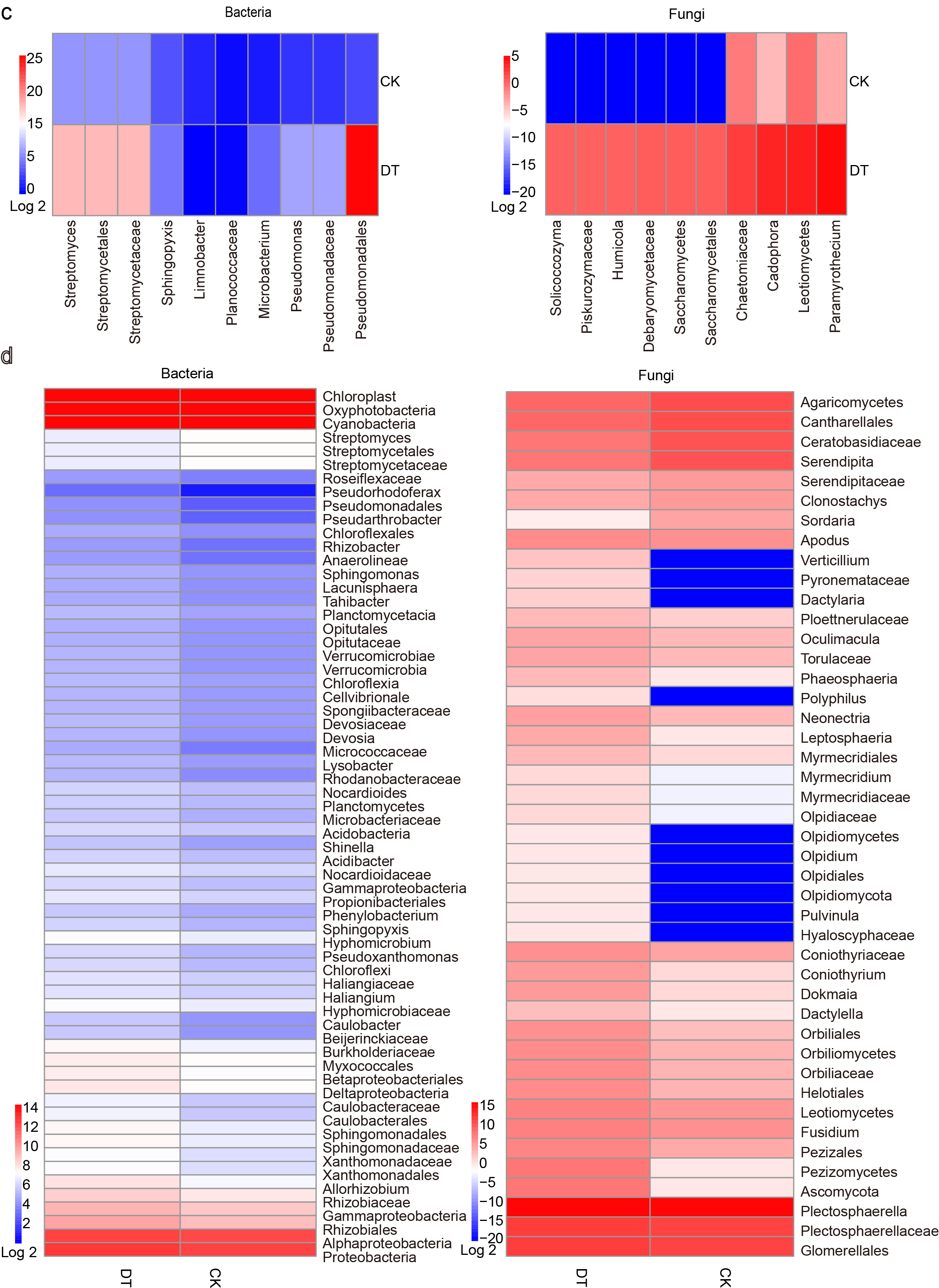
**

**Fig. S1** The abundances of enriched taxa in *C. pilosula* parts

(a): The abundances of enriched taxa in different parts of *C. pilosula*. (b), (c), and (d): The abundances of enriched taxa in leaf (b), stem (c), and root (d) parts of *C. pilosula* under drought stress. CK, untreated group; DT, drought treatment group.

**Table S1** **Numbers and average lengths of microbial sequences** **in leaf part of *C. pilosula*.**

|  | bacterial community | | | fungal community | | |
| --- | --- | --- | --- | --- | --- | --- |
|  | sequence | bases (bp) | average length (bp) | sequence | bases (bp) | average length (bp) |
|  |  |  |  |  |  |  |
| DT1- rep 1 | 51,519 | 19,404,647 | 377 | 62,506 | 14,849,637 | 238 |
| DT1- rep 2 | 72,610 | 27,352,744 | 377 | 65,904 | 15,864,144 | 241 |
| DT1- rep 3 | 51,122 | 19,257,068 | 377 | 51,780 | 12,341,065 | 238 |
| CK1- rep 1 | 51,332 | 19,334,679 | 377 | 56,281 | 14,373,328 | 255 |
| CK1- rep 2 | 57,990 | 21,842,431 | 377 | 51,044 | 12,578,675 | 246 |
| CK1- rep 3 | 67,143 | 25,288,127 | 377 | 56,545 | 13,258,294 | 234 |
| DT2- rep 1 | 54,635 | 20,567,077 | 376 | 60,289 | 14,367,075 | 238 |
| DT2- rep 2 | 54,940 | 20,681,704 | 376 | 62,487 | 14,862,725 | 238 |
| DT2- rep 3 | 67,757 | 25,512,278 | 377 | 58,108 | 13,886,249 | 239 |
| CK2- rep 1 | 71,630 | 26,972,087 | 377 | 66,663 | 16,155,658 | 242 |
| CK2- rep 2 | 70,950 | 26,712,977 | 377 | 73,588 | 17,744,959 | 241 |
| CK2- rep 3 | 69,544 | 26,183,206 | 377 | 70,011 | 16,884,942 | 241 |
| DT3- rep 1 | 61,944 | 23,320,076 | 376 | 54,953 | 12,922,051 | 235 |
| DT3- rep 2 | 67,274 | 25,320,741 | 376 | 51,531 | 12,073,307 | 234 |
| DT3- rep 3 | 62,072 | 23,366,255 | 376 | 52,398 | 12,368,518 | 236 |
| CK3- rep 1 | 67,697 | 25,482,860 | 376 | 67,587 | 16,112,397 | 238 |
| CK3- rep 2 | 57,306 | 21,577,680 | 377 | 63,159 | 15,135,852 | 240 |
| CK3- rep 3 | 72,357 | 27,254,777 | 377 | 57,853 | 13,851,489 | 239 |
| Average | 62,768 | 23,635,079 | 377 | 60,149 | 14,423,909 | 240 |

CK, untreated group; DT, drought treatment group; DT1, DT3, and DT8 represented the treatment group on days 1, 3, and 8 of drought stress, respectively; CK1, CK3, and CK8 represented the untreated group on days 1, 3, and 8 of drought stress, respectively. rep, biological repeat.

**Table S2** Numbers and average lengths of microbial sequences in stem part of *C. pilosula*.

|  | bacterial community | | | fungal community | | |
| --- | --- | --- | --- | --- | --- | --- |
|  | sequence | bases (bp) | average length (bp) | sequence | bases (bp) | average length (bp) |
|  |  |  |  |  |  |  |
| DT1- rep 1 | 63,158 | 23,734,920 | 376 | 52,406 | 11,861,463 | 226 |
| DT1- rep 2 | 71,271 | 26,782,085 | 376 | 73,414 | 16,568,325 | 226 |
| DT1- rep 3 | 67,479 | 25,358,803 | 376 | 64,144 | 14,480,400 | 226 |
| CK1- rep 1 | 64,907 | 24,380,762 | 376 | 67,508 | 15,056,029 | 223 |
| CK1- rep 2 | 60,365 | 22,684,585 | 376 | 55,986 | 12,484,306 | 223 |
| CK1- rep 3 | 71,957 | 27,040,910 | 376 | 53,831 | 12,004,129 | 223 |
| DT2- rep 1 | 68,941 | 25,923,459 | 376 | 54,431 | 12,136,929 | 223 |
| DT2- rep 2 | 62,224 | 23,398,383 | 376 | 51,216 | 11,421,509 | 223 |
| DT2- rep 3 | 51,076 | 19,203,567 | 376 | 70,163 | 15,649,171 | 223 |
| CK2- rep 1 | 52,706 | 19,813,130 | 376 | 52,420 | 11,714,693 | 223 |
| CK2- rep 2 | 71,456 | 26,848,186 | 376 | 64,505 | 14,421,077 | 224 |
| CK2- rep 3 | 54,465 | 20,464,048 | 376 | 58,449 | 13,080,811 | 224 |
| DT3- rep 1 | 72,710 | 27,296,418 | 375 | 51,296 | 11,421,112 | 223 |
| DT3- rep 2 | 66,741 | 25,070,272 | 376 | 73,713 | 16,368,201 | 222 |
| DT3- rep 3 | 69,677 | 26,182,136 | 376 | 51,757 | 11,510,046 | 222 |
| CK3- rep 1 | 61,565 | 23,145,260 | 376 | 60,552 | 13,651,060 | 225 |
| CK3- rep 2 | 55,850 | 20,990,893 | 376 | 53,305 | 11,990,237 | 225 |
| CK3- rep 3 | 54,425 | 20,460,715 | 376 | 51,224 | 11,555,341 | 226 |
| Average | 63,387 | 23,821,030 | 376 | 58,907 | 13,187,491 | 224 |

Abbreviations such as on previous table.

**Table S3** Numbers and average lengths of microbial sequences in root part of *C. pilosula*

|  | bacterial community | | | fungal community | | |
| --- | --- | --- | --- | --- | --- | --- |
|  | sequence | bases (bp) | average length (bp) | sequence | bases (bp) | average length (bp) |
|  |  |  |  |  |  |  |
| DT1- rep 1 | 65,475 | 24,537,364 | 375 | 58,736 | 13,332,391 | 227 |
| DT1- rep 2 | 74,676 | 27,996,138 | 375 | 65,361 | 14,975,377 | 229 |
| DT1- rep 3 | 64,946 | 24,355,130 | 375 | 53,539 | 12,232,004 | 228 |
| CK1- rep 1 | 58,341 | 21,889,512 | 375 | 74,508 | 16,940,447 | 227 |
| CK1- rep 2 | 71,830 | 26,960,182 | 375 | 71,112 | 16,236,606 | 228 |
| CK1- rep 3 | 59,005 | 22,135,844 | 375 | 59,326 | 13,500,843 | 228 |
| DT2- rep 1 | 65,377 | 24,525,703 | 375 | 52,448 | 11,842,610 | 226 |
| DT2- rep 2 | 71,073 | 26,653,706 | 375 | 66,708 | 15,038,033 | 225 |
| DT2- rep 3 | 63,275 | 23,738,779 | 375 | 61,884 | 13,962,227 | 226 |
| CK2- rep 1 | 55,239 | 20,736,652 | 375 | 70,986 | 15,993,209 | 225 |
| CK2- rep 2 | 73,295 | 27,500,777 | 375 | 60,784 | 13,685,058 | 225 |
| CK2- rep 3 | 71,009 | 26,650,146 | 375 | 69,919 | 15,757,657 | 225 |
| DT3- rep 1 | 61,336 | 23,028,825 | 375 | 71,480 | 16,403,474 | 229 |
| DT3- rep 2 | 52,220 | 19,598,798 | 375 | 62,082 | 14,250,333 | 230 |
| DT3- rep 3 | 50,338 | 18,901,161 | 375 | 56,617 | 12,937,496 | 229 |
| CK3- rep 1 | 53,062 | 19,910,227 | 375 | 62,343 | 14,356,008 | 230 |
| CK3- rep 2 | 58,735 | 22,031,206 | 375 | 60,734 | 14,057,488 | 231 |
| CK3- rep 3 | 52,278 | 19,599,895 | 375 | 71,182 | 16,489,525 | 232 |
| Average | 62,306 | 23,375,003 | 375 | 63,875 | 14,555,044 | 228 |

cAbbreviations such as on previous table.
